# A Case of Eosinophilic Angiocentric Fibrosis With Palatal Fistulas

**DOI:** 10.7759/cureus.30938

**Published:** 2022-10-31

**Authors:** Vaibhav R Kadam, Andrew Vaughn

**Affiliations:** 1 Otolaryngology, University of Pikeville - Kentucky College of Osteopathic Medicine, Pikeville, USA; 2 Otolaryngology, Lake Cumberland Regional Hospital, Somerset, USA

**Keywords:** igg4-related disease, septal perforation, perivascular fibrosis, eosinophils, oronasal fistula, eosinophilic angiocentric fibrosis

## Abstract

Eosinophilic angiocentric fibrosis (EAF) is a rare, but benign, tumefactive lesion of the head and neck regions. It was initially discovered in 1983 but has recently been connected to the spectrum of immunoglobulin G4-related disease (IgG4-RD). It commonly presents with symptoms of nasal obstruction, structural deformities of the external nose, and involvement of the nasal septum and lateral nasal wall. Our patient presented with a saddle nose deformity, a septal perforation, and palatal fistulas. Laboratory testing for EAF is often negative for the presence of antinuclear cytoplasmic antibodies (ANCA). A definitive diagnosis of EAF can be made through histopathological analysis of the lesion. The appearance of "onion-skin" fibrosis with perivascular infiltration of primary eosinophils is pathognomonic for EAF. While there is a presence of ulceration tissue, EAF does not have any histological signs of necrosis. EAF is a very uncommon etiology of nasal obstructive symptoms; therefore, it is necessary to rule out more conventional pathologies. Even though it appears as a malignant process, it has an excellent prognosis. The common treatment modalities for an active lesion of EAF involve either surgical resection of margins alone or a combination of corticosteroids and resection. Rituximab has also shown benefits in the management of IgG4-RD as a corticosteroid-sparing treatment. Rituximab was chosen for treatment in our patient because surgical resection was not possible due to the absence of an active lesion. In this article, we provide a brief review of EAF and provide a unique case of EAF presenting with oronasal palatal fistulas.

## Introduction

Eosinophilic angiocentric fibrosis (EAF) is a unique condition of undetermined etiology resulting in a chronic inflammatory process often found in the head and neck regions. As of 2018, there have been 54 cases reported in the literature [1]. It is a fairly new disease process that was first identified in 1983 and has been accepted into the spectrum of immunoglobulin G4-related disease (IgG4-RD) more recently based on the common histopathological findings [1,2]. It is a diagnosis that must be made upon biopsy findings consistent with an eosinophilic inflammatory infiltrate with fibrosis and accentuation around blood vessels forming a classic "onion-skin" pattern of fibrosis [3]. A lymphoplasmacytic infiltrate consisting of immunoglobulin G (IgG) and immunoglobulin G4 (IgG4)-positive plasma cells can also be found linking EAF to the IgG4-RD spectrum [2]. Here, we describe a unique case of EAF initially presenting with soft and hard palate oronasal fistulas along with a history of chronic upper respiratory symptoms.

This article was previously presented as a poster at the Annual University of Pikeville Research Symposium on April 8, 2022.

## Case presentation

**Figure 1:**
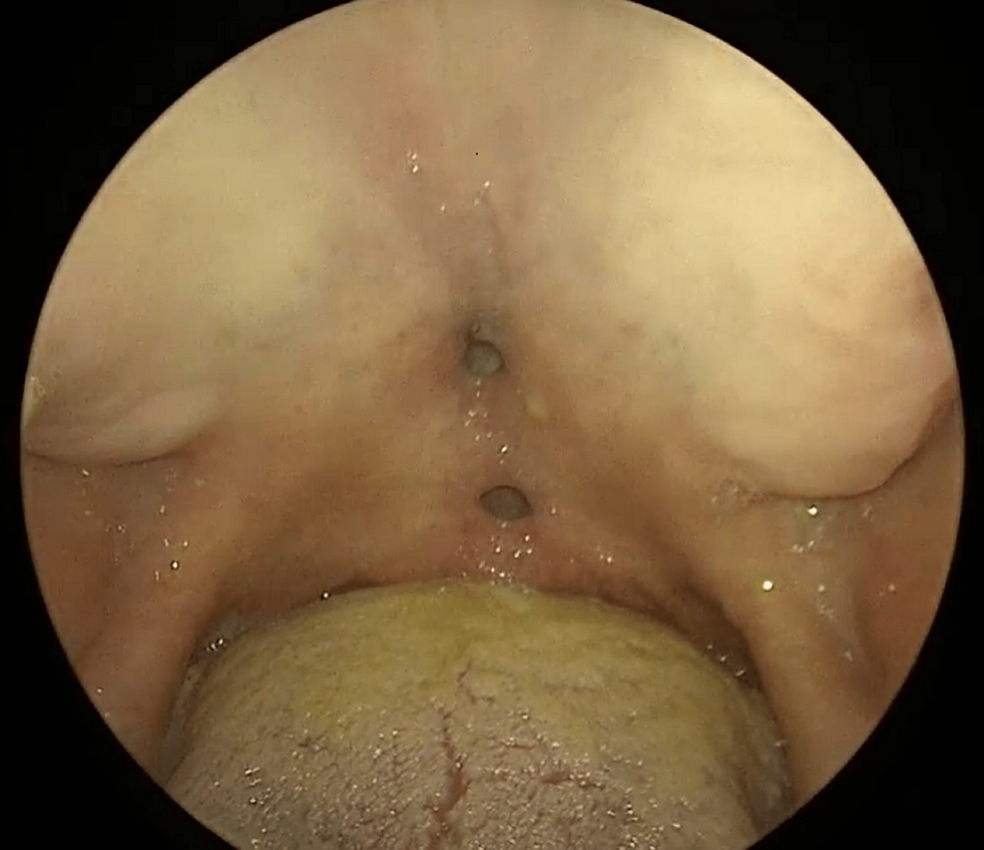
Oronasal palatal fistulas

A 41-year-old female presented to the otolaryngology office for evaluation of a soft palate fistula. She presented with one-year-long complaints of purulent and serosanguineous nasal secretions, which have been refractory to numerous courses of antibiotics. She also reports a one-year-long alteration in the appearance of her external nose leading to a saddle nose deformity. Her past medical history is notable for rheumatoid arthritis (RA), which is currently managed with infliximab, and she denied any history of chronic allergies. Upon interviewing the patient, she was found to have a hypernasal quality in her speech. A single hard palate oronasal fistula and a single soft palate oronasal fistula were present on the physical exam (Figure 1).

**Figure 2:**
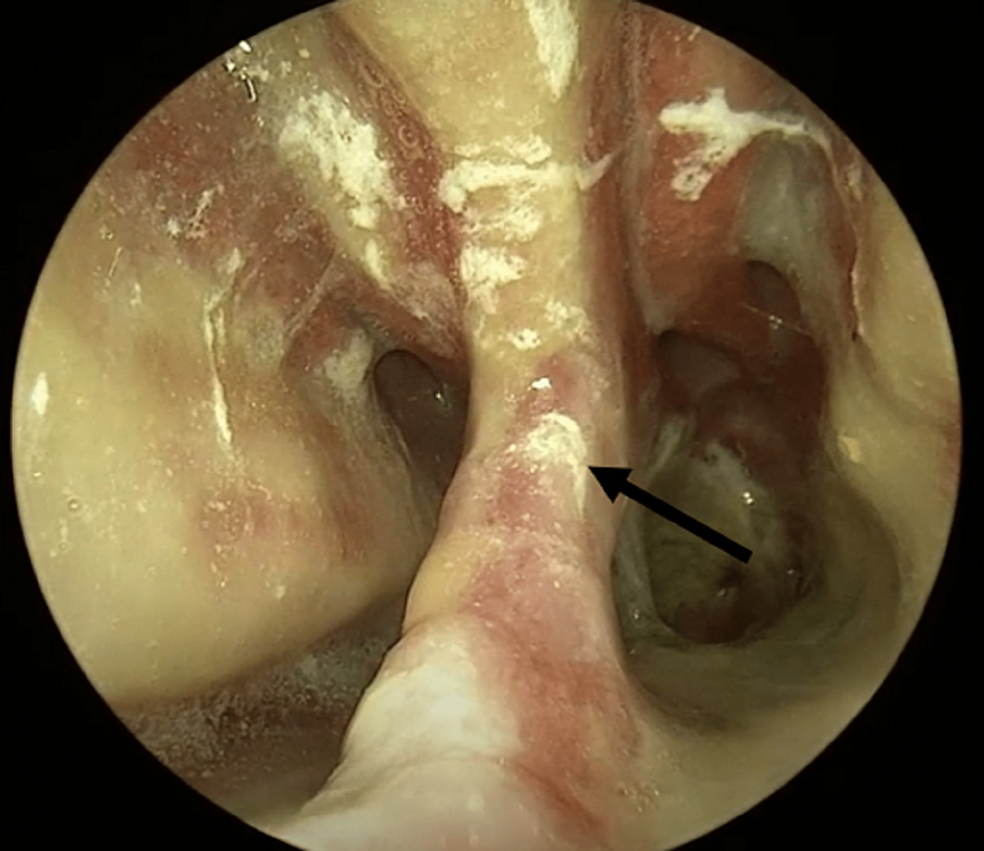
Nasal septal perforation view from left nares

The presence of a saddle nose deformity and bilateral distal nasal canal dry secretions prompted further nasal evaluation via a 0-degree endoscope. An examination of her neck found no palpable masses or lymphadenopathy. Nasal endoscopy found a large midline septal perforation (Figure 2).

No hypertrophy or edema of the turbinates was found bilaterally. The presence of septal erosion raised concerns for granulomatosis with polyangiitis (GPA) or eosinophilic granulomatosis with polyangiitis (EGPA) leading to subsequent laboratory studies for the presence of antinuclear cytoplasmic antibodies (ANCA). Her antinuclear antibody (ANA) titer was greater than 1:80. The cytoplasmic-antinuclear cytoplasmic antibody (c-ANCA), perinuclear-antinuclear cytoplasmic antibody (p-ANCA), antimyeloperoxidase (MPO) antibody, antiproteinase-3 (PR-3) antibody, and atypical p-ANCA titers were all found to be negative, which indicated a need for further workup.

**Figure 3:**
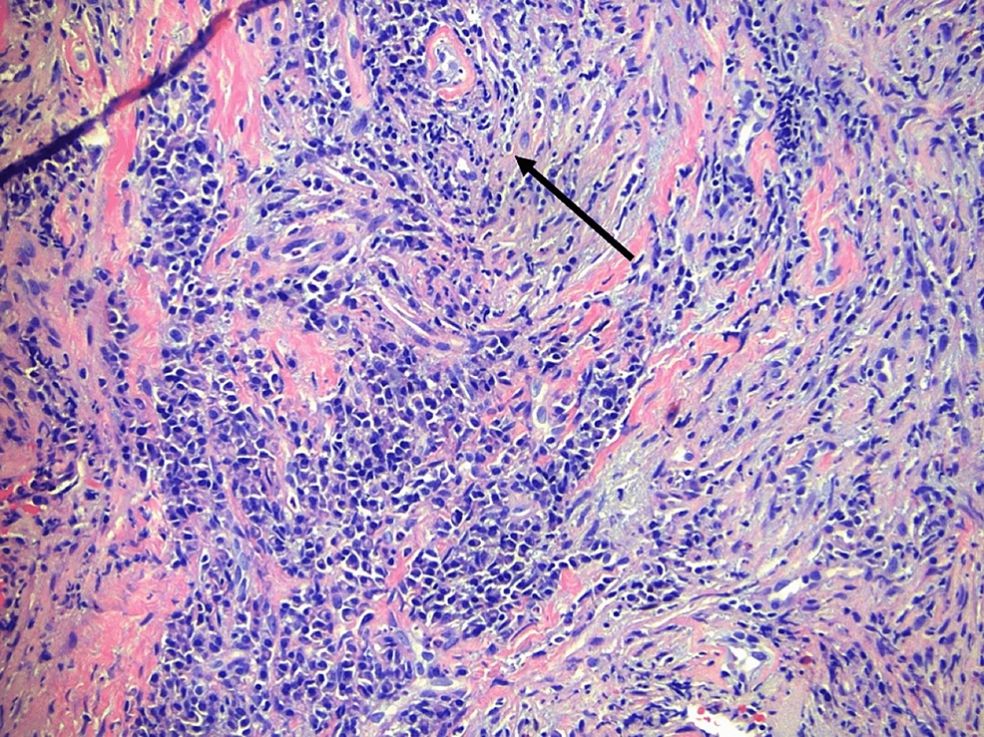
Perivascular fibroinflammatory lesion with abundant plasma cells, lymphocytes, eosinophils, and histiocytes on hematoxylin and eosin stain

The patient presented for a one-week follow-up with reports of continued hypernasal speech, purulent nasal discharge, and increased difficulty swallowing. Repeat nasal endoscopy exhibited similar results. However, there was purulent drainage noted in the middle nasal meatus bilaterally. The patient was scheduled for a nasal biopsy to be performed in the operating room. Prior to the procedure, a bilateral sino-nasal fungal culture swab was performed to rule out fungal infection. The results showed the presence of *Candida dubliniensis *(*C. dubliniensis*) and *Candida glabrata *(*C. glabrata*). Three specimens were collected from the nasal biopsy: posterior nasal septum (5 mm x 5 mm), left inferior turbinate (5 mm x 5 mm), and right inferior turbinate (10 mm x 5 mm x 2 mm). All three specimens showed similar histological findings with a mixture of chronic inflammation with abundant plasma cells, lymphocytes, eosinophils, and histiocytes (Figure 3).

The biopsy also contained dense fibrosis with hyalinization and accentuation around blood vessels, nerves, and areas of mucosal ulceration. Immunohistochemical staining primarily discovered cluster of differentiation 3 (CD3)-positive T-cells, CD20-positive B-cells, and IgG-positive plasma cells. No convincing signs of vasculitis were seen in the specimens. However, IgG immunostaining revealed extensive positivity in the plasma cells, while IgG4 immunostaining exhibited up to 40 IgG4 plasma cells per high-power field. With the absence of a neoplastic hematolymphoid process, the pathological findings are most consistent with a fibroinflammatory process. The presenting fibrosis combined with the presence of IgG4 plasma cells points toward a diagnosis of EAF. With no evidence of giant cells or convincing signs of vasculitis, it argues against the differential of GPA. Her EAF is being managed in conjunction with her rheumatologist who has begun treatment with rituximab.

## Discussion

EAF has often been described as a distinctive perivascular fibroinflammatory process commonly found to affect the nasal septum and surrounding structures [4]. Depending on the structures that are affected, patients can present with a variety of initial complaints, primarily nasal obstruction followed by a change in external nasal appearance [5]. To our knowledge, EAF has been reported in the literature with the involvement of the nasal septum, paranasal sinuses, and orbit, but it has not been seen with oronasal fistulas [3-8]. Instead of the common finding of nasal obstruction, our patient presented with velopharyngeal insufficiency secondary to her oronasal fistulas. This unique presentation can be attributed to EAF, which commonly causes local tissue destruction, particularly of the nasal septum [6]. In addition, the alternation of our patient’s nose to a saddle nose deformity within a one-year time span is consistent with the physical findings of EAF. Nasal septum and lateral wall involvement are shared characteristics in patients with EAF along with pathology consistent with ulceration but lacking evidence of necrosis [7]. The physical exam findings of our patient are highly suggestive of EAF.

A definitive diagnosis of EAF is determined through histopathological findings. The criteria pathognomonic for EAF is the histological appearance of a "storiform" pattern of fibrosis with perivascular infiltration of polymorphonuclear leukocytes consisting primarily of eosinophils [1]. With the increased number of eosinophils seen on histology, it is important to evaluate for a history of chronic allergies. Our patient denied any history of chronic allergies, and a predisposing allergic etiology is not required to rule in the diagnosis [4]. More recently, EAF has been included in the category of IgG4-RD based on histological criteria of a lymphoplasmacytic infiltrate. The presence of greater than 50 IgG4-positive plasma cells per high-power field has been noted to have high specificity for confirmation of IgG4-RD [9]. We did not perform serological immunoglobulin testing on our patient. While our pathology results showed extensive IgG- and IgG4-positive plasma cells, they did not meet the specific criteria to be considered in the category of IgG4-RD.

Prior to reaching a diagnosis of EAF, it is essential to rule out differential etiologies that present as destructive nasopharyngeal lesions. The vasculitides, specifically GPA, should always be considered when evaluating patients with abnormalities such as saddle nose deformities and persistent upper respiratory symptoms [10]. Laboratory testing for antineutrophil cytoplasmic antibodies for ANCA-associated vasculitides can be done via immunofluorescence assays (IFA) or enzyme-linked immunosorbent assays (ELISA) [11]. It is important to note that the positivity of ANCA testing is reliant upon the clinical manifestations of disease in the patient; therefore, a patient presenting with a multitude of symptoms related to a vasculitic process is more likely to have a positive titer compared to a patient with minimal manifestations [12]. The combination of negative ANCA studies and physical findings corresponding with a vasculitic process should raise suspicion for EAF. Biopsy results are another vital component to evaluate for vasculitis. Cutaneous vasculitis is commonly described to have a process of perivascular fibrinoid necrosis with infiltration of polymorphonuclear neutrophils into the vessel walls [13]. While the biopsy results showed a similar angiocentric pattern, the lack of granuloma formation and tissue necrosis argues against a diagnosis of vasculitis. In other cases of diagnosed EAF, both ANA and ANCA titers were found to be negative [3,6,8]. The ANA titer of greater than 1:80 in our patient is mostly due to her long-standing history of RA [14].

We must also take into account our patient’s medical history of RA when discussing differentials of EAF. With her treatment regimen consisting of immunosuppressive therapy, opportunistic pathogens could be considered a possible cause of her symptoms. With evidence for a septal perforation, angioinvasive fungal sinusitis should be ruled out as it commonly manifests with tissue and vessel invasion leading to necrosis of the nasopharyngeal structures [15]. Fungal cultures were not positive for invasive fungal species but did show the presence of *C. glabrata* and *C. dubliniensis*. *C. dubliniensis* is commonly found in immunosuppressed patients, but in the absence of signs of systemic candidemia, it is an incidental finding [16]. It is no surprise that *C. glabrata* is present in the nares because it is a nonpathogenic organism part of the normal human flora [17]. In the absence of evidence for an angioinvasive fungal process, further investigation was necessary to reach the diagnosis of EAF.

Due to the limited amount of data available, there are no specific therapeutic guidelines for the management of EAF. The treatment modality for managing EAF indicates a surgical approach over medical management. The ideal technique involves surgical excision of the margins or debulking surgery [18]. In a systematic review by Fang et al., surgical resection was the primary treatment modality followed by a combination of resection and corticosteroid use [19]. Total resection of the affected tissues is an important role to prevent the recurrence of the disease [6]. EAF is seen to have an excellent prognosis following either treatment as 100% of patients were alive at scheduled follow-up visits [19]. Currently, our patient is being managed with an intravenous infusion of rituximab ordered by her rheumatologist for corticosteroid-sparing treatment because no active lesion is present for resection. Rituximab has been shown to improve serological markers as well as the destructive lesions of IgG4-RD [2]. Rituximab is the treatment of choice for the management of IgG4-RD because it leads to a decline in the production of IgG4-positive plasma cells responsible for the histopathological features seen in EAF [20].

## Conclusions

In conclusion, EAF is a rare, but benign, lesion of unknown etiology involving the upper respiratory tract. It commonly presents with nasal obstruction and external nose deformity, but in our case, it initially presented with oronasal fistulas and velopharyngeal insufficiency. It is crucial to rule out other etiologies of destructive nasal lesions such as vasculitides or fungal infection prior to arriving at the diagnosis of EAF. A histopathological analysis is necessary to ascertain the diagnosis of EAF, which shows an eosinophilic infiltrate with a dense "onion-skin" pattern of fibrosis. With its recent introduction into the spectrum of IgG4-related disease, the presence of IgG- and IgG4-positive plasma cells should be taken into account. The management of EAF can include surgical resection or surgical resection with corticosteroids for active lesions. It can also be managed with rituximab for serological and clinical improvement in the absence of an active lesion. We hope to shed more light on this atypical pathology with a unique presentation of soft and hard palatal oronasal fistulas.
